# Multiple Pulse Amperometry—An Antifouling Approach for Nitrite Determination Using Carbon Fiber Microelectrodes

**DOI:** 10.3390/molecules28010387

**Published:** 2023-01-02

**Authors:** Douglas P. M. Saraiva, Daniel V. Braga, Bruna Bossard, Mauro Bertotti

**Affiliations:** Department of Fundamental Chemistry, Institute of Chemistry, University of São Paulo, Av. Prof. Lineu Prestes, 748, São Paulo 05513-970, SP, Brazil

**Keywords:** nitrite, pulsed amperometry, electroanalysis, antifouling

## Abstract

Nitrite is a ubiquitous pollutant in modern society. Developing new strategies for its determination is very important, and electroanalytical methods present outstanding performance on this task. However, the use of bare electrodes is not recommended because of their predisposition to poisoning and passivation. We herein report a procedure to overcome these limitations on carbon fiber microelectrodes through pulsed amperometry. A three-pulse amperometry approach was used to reduce the current decay from 47% (after 20 min under constant potential) to virtually 0%. Repeatability and reproducibility were found to have an RSD lower than 0.5% and 7%, respectively. Tap water and synthetic inorganic saliva samples were fortified with nitrite, and the results obtained with the proposed sensor were in good agreement with the amount added.

## 1. Introduction

Nitrite is widely used as a coloring agent [1], food additive [2,3], and preservative [4,5]. In the chemical industry, it is a major precursor to nitro-rubber production [6]. In the form of sodium nitrite, it is also used as a grease additive, acting as a corrosion inhibitor [7]. In the food industry, nitrite is used as a preservative because it is a lipid peroxidation retardant and has antimicrobial properties and the capability to retard rancidity during storage [4,5]. In meat products, nitrite is employed as a flavoring and coloring agent [1,3].

Nitrite is found everywhere in modern society, and unfortunately, it is also a pollutant and has diverse implications on animal and human health and the surrounding environment [8]. To human health, nitrite can interact with hemoglobin oxidizing the ferrous iron center into its ferric state forming the methemoglobin which inhibits oxygen transport [9]. Nitrite can also interact with amines to form N-nitrosamines, which the International Association for Research on Cancer mentions as carcinogenic [10]. Hence, according to the World Health Organization (WHO), the guideline value for nitrite in drinking water is 3 mg L^−1^ as nitrite ion [11] and the US-FDA guideline for nitrite in meat products is 200 ppm [12].

Due to the high risk to human health and environmental problems, many methods have been described for sensitive nitrite detection and quantification. Common methods for nitrite quantification include electrophoresis [13], chromatography [14,15], chemiluminescence [16], and spectrophotometry [14,17]. Despite the selectivity and sensitivity of some of these methods, there are some limitations and drawbacks, such as narrow application ranges, complex sample treatment, impossibility of real-time application, and difficulted miniaturization [18].

Thus, compared to these methods, electrochemical detection of nitrite has advantages, such as cost-effectiveness, ease of operation, ease of miniaturization, real-time detection, simple sample treatment, and a wide linear detection range [19]. The detection of nitrite through electrochemical methods can be accomplished by reduction or oxidation, although the detection based on the reduction process usually has a major interference from oxygen and nitrate. Then, the preferred detection method is through nitrite oxidation, alleviating problems with some common interferents [20,21].

Although electrochemical detection via oxidation is usually preferred, the use of bare electrodes is limited by surface poisoning and passivation [22,23]. Some strategies can be employed to overcome these limitations, such as electrode surface modification, pretreatment [24], and pre-stabilizing the electrode signal before the analysis. The modification of the electrode surface has been shown to enhance the signal stability significantly, even though the mechanism associated with the antifouling effect still needs to be fully elucidated [25,26].

Some electrodes described in the literature suffer from signal loss during the first minutes and hours. These sensors usually need a long stabilization period before use. An emblematic example is the WPI electrodes (World Precision Instruments Inc., Sarasota, FL, USA) for nitric oxide detection, which require a stabilization time of about two hours before usage [27]. Another common strategy is the pretreatment of the electrode surface by polarizing the electrode at positive or negative potentials over a few minutes before the measurements [24,28].

A natural advance from the pretreatment strategy is multiple pulse amperometry (MPA). In this technique, a brief potential pulse can reactivate the electrode surface every few seconds, while another pulse can be used for analyte detection. In such an approach, third and fourth potential pulses can stabilize the background current, remove interferents contributions, or analyze a second analyte [29]. This technique has been successfully used for various analytes such as ascorbic acid [30], paracetamol [30,31], caffeine [29,31], ibuprofen [31], sulfide [32], and nitrite [32,33].

In this work, we show our efforts to diminish electrode poisoning in a carbon fiber microelectrode without further modification by using a pulsed amperometric method. Such an approach reactivates the surface of the carbon fiber microelectrode and was shown to be helpful in enhancing the signal and the response stability toward nitrite detection.

## 2. Results and Discussion

**Figure 1:**
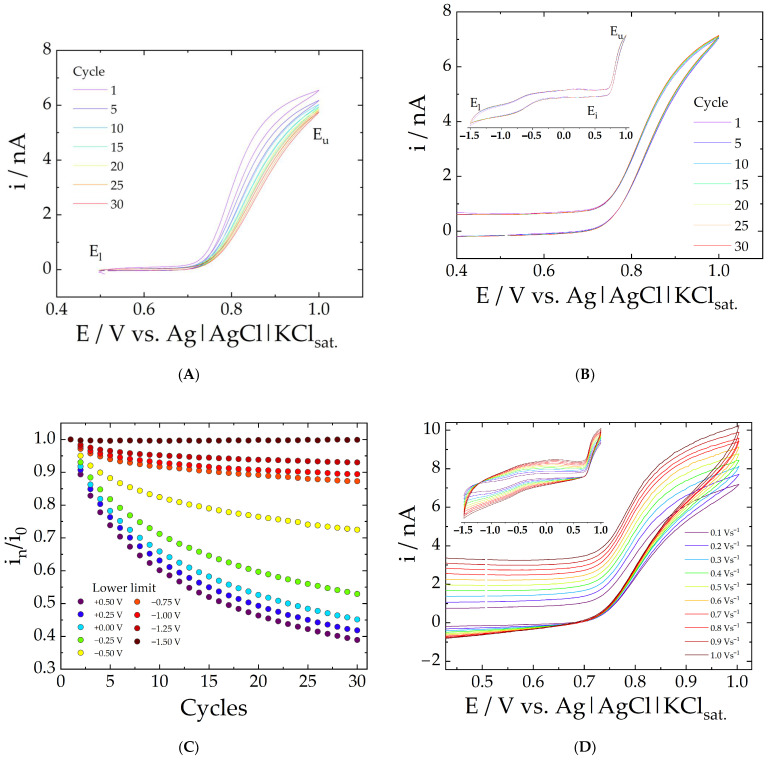
Consecutive cyclic voltammograms (n = 30) recorded with a carbon fiber microelectrode in PBS containing 2 mmol L^−1^ nitrite (scan rate = 100 mV s^−1^) from the initial potential (E_i_ = 0.50 V) to varying lower limit switching potentials (E_l_ = + 0.50 V (**A**) and −1.50 V (**B**)), then to the upper limit switching potential (E_u_ = 1.00 V), and then back to E_i_. (**C**) shows current loss values (i_n_ / i_o_, where i_o_ corresponds to the first scan) measured at 0.90 V as a function of the number of cycles (n) for different lower limit switching potentials (E_l_). (**D**) shows the 30th-cycle recorded in PBS containing 2 mmol L^−1^ nitrite at various scan rates.

The nitrite oxidation signal recorded by cyclic voltammetry with a freshly polished electrode decays at each cycle, and the passivation of the surface by oxides produced during nitrite oxidation [34] or poisoning of the electrode usually explains this response loss [22]. Figure 1A shows an example of such a problem, as a continuous anodic current decrease is observed during the potential cycling using a carbon fiber microelectrode. The current decrease in the CVs, at 0.825V, is about 2 nA after 30 cycles, and this can only be explained by the passivation or poisoning of the electrode surface. It should be pointed out that the current decrease in CVs recorded in PBS using the same conditions at 0.825V is only about 10 pA (Supporting Information, Figure S1). This confirms that the poisoning of the electrode surface is attributed to nitrite oxidation products. As expected, such a signal decay over time represents a major issue for any analytical application.

This electrode surface poisoning, however, can be alleviated by an unconventional potential application program involving polarization of the electrode at suitable potentials. In this first section, we bring the first evidence that the signal stability can be enhanced by polarizing the electrode at very negative potentials.

The usefulness of the proposed protocol can be noticed by recording consecutive cyclic voltammograms in nitrite solution from the initial potential (E_i_ = 0.50 V) to a lower switching potential (E_l_ = −1.50 V), then to the upper limit switching potential (E_u_ = 1.00 V). The superimposed voltammograms, seen in Figure 1B, are clear evidence that a fresh and reproducible surface is obtained at each new cycle. Figure 1C shows the results of a systematic study where different lower limit switching potentials (E_l_) were employed. The response loss is represented by the i_n_/i_o_ ratio, which corresponds to the current value measured at 0.90 V normalized by the current in the first scan. The continuous response loss during the voltammograms recording is much more important at less negative lower switching potential values (E_l_), and no significant change is observed at E_l_ = −1.50 V after 30 potential cycles.

Although signal stability can be achieved in voltammetric conditions, the data collection frequency depends on the scan rate. At the conditions presented in Figure 1B, each cycle takes about 50 s to be completed, and the data collection frequency is very low. If one is to enhance the data collection frequency by increasing the scan rate, the electrical double-layer effect would be detrimental to the measurements, interfering with the background noise and blank signal, as can be seen in Figure 1D. Pulsed amperometric techniques can be used to overcome these limitations; hence, further experiments were based on pulsed amperometry and the aforementioned observations.

### 2.1. Amperometric Reactivation Method Optimization

The proposed protocol relies on a three-pulse amperometric method: (**i**) a negative potential pulse for the electrodic surface regeneration (E_l_); (**ii**) a stabilizing potential pulse whose value is less negative than the one required for analyte oxidation, but more positive than those of possible interfering compounds; and (**iii**) a third potential pulse for the analyte oxidation. The Faradaic response involving the target analyte corresponds to the subtraction of current values measured at the third pulse and the stabilizing one. This approach is particularly useful in the case of complex samples, as the current component corresponding to the interferences can be discounted.

In this three-pulse amperometric method, both the potential and step time are variables prone to optimization, and as such, experiments were carried out to investigate the influence of such experimental parameters on the response regarding the nitrite anodic oxidation process. As previously demonstrated in the voltammetric experiments (Figure 1), the reactivation potential (E_r_) was found to exert an important role in signal stability. Accordingly, a reactivation potential of −1.50 V (E_r_ = −1.50 V), and time step of 0.2 s allowed nitrite oxidation current to present no significative variation (Supporting Information, Figure S2A).

The step time influence was also examined in the 0.1 to 1.0 s range (at E_r_= −1.50 V), and the results regarding the nitrite oxidation (measured at 0.90 V) yielded a Vulcan graph type. However, less stable responses were observed for step times higher than 0.3 s, and under these circumstances, the best parameters for reactivation potential and step time were found to be 0.2 s at −1.50 V (Supporting Information, Figure S2B).

The parameters associated with the stabilizing potential pulse were also investigated considering the nature of the sample. For instance, more negative potentials are preferred for clean samples containing no detectable interfering compounds, Figure S3A. On the other hand, if the sample does contain interferents that undergo oxidation at less positive potentials than the analyte, the stabilizing potential must be chosen in a potential more positive than the one corresponding to the interferent.

The stabilizing pulse step must also be observed with caution. The longer the step time, the smaller its influence on the final response, which corresponds to the subtraction of current values measured in the oxidation pulse and the stabilizing one, Figure S3B. The duration of the stabilizing pulse, however, cannot be increased indefinitely, since a longer duration implies a higher interval time between data points, which can be detrimental to methods such as FIA and BIA. For experiments without detectable interferents, the best parameters for the stabilizing step were found to be 1.4 s at 0.50 V.

Finally, the influence of the nitrite oxidation potential and the step time were also studied considering the electrochemical behavior of the target analyte at the carbon fiber microelectrode surface. For instance, more positive potentials will induce faster signal loss owing to the formation of oxidized species that adsorb and poison the electrode surface. On the other hand, the reaction must be driven at mass transport conditions to avoid the kinetic regime and to increase the sensitivity. Similar principles are applied to the oxidation pulse step, i.e., short values will reduce the passivation and contribute to signal stability and repeatability. However, very short oxidation pulses should be avoided because of the relatively large contribution of the capacitive component. The best compromise was achieved for E = 0.90 V and step time = 0.2 s (Supporting Information, Figure S4).

### 2.2. Signal Stability Comparison Using Different Techniques

**Figure 2:**
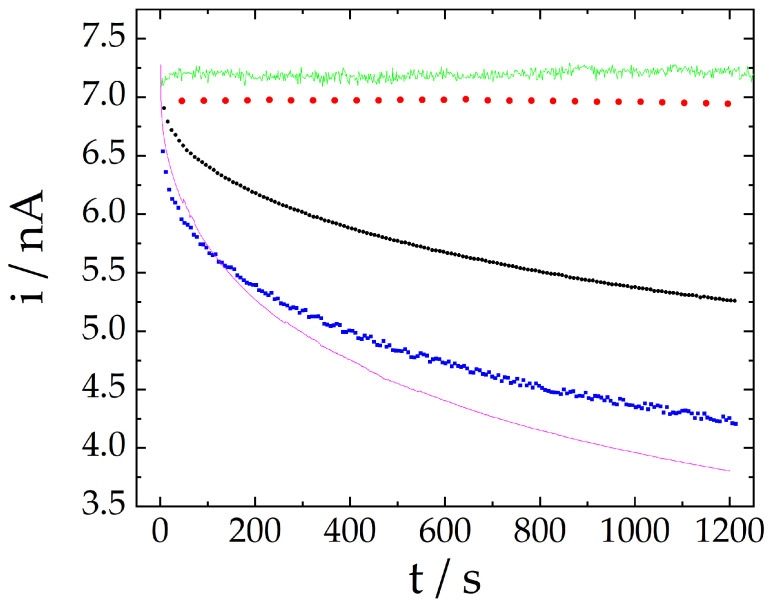
Current responses measured in a 2.0 mmol L^−^^1^ nitrite solution by cyclic voltammetry (in black, 100 mV s^−^^1^), cyclic voltammetry with extended potential range (in red, 100 mV s^−^^1^), differential pulse voltammetry (in blue), amperometry (in pink) and the proposed method (three-pulse amperometric method, in green). Current values for cyclic voltammetry and for differential pulse voltammetry correspond to the signal at E= 0.90 V and at the peak potential (first repetition, 20 min), respectively.

Figure 2 brings a comparison between the signal stability regarding the nitrite anodic response using cyclic voltammetry, cyclic voltammetry with extended potential range, differential pulse voltammetry, amperometry, and the proposed method (three-pulse amperometric method). The more significant signal loss was noticed for common amperometry, at around 47% after 20 min. For microelectrodes with very small radius, a stationary current is expected to be achieved at short times (milliseconds range) because of the radial diffusion. Hence, the continuous current decrease is not associated with the expansion of the diffusion layer and depletion of the analyte near the electrode surface but is rather the result of the poisoning and passivation effect. The data collected from cyclic voltammetry and differential pulse voltammetry also demonstrated a continuous current decrease as a consequence of the accumulation of nitrogen oxides onto the surface of the microelectrode surface.

On the other hand, better results were obtained using voltammetry with the potential extension up to −1.50 V because the electrode surface is renewed each cycle. Results were even improved in terms of sensitivity and stability by using the proposed approach, as a signal change of less than 0.9% was noticed after 20 min. It should be pointed out that the noise seems to be inherent to the technique, as already reported in the literature for detection with double pulse amperometry (DPA) and multiple pulse amperometry (MPA) [29]. Table 1 summarizes the stability test results for the aforementioned techniques.

The signal stabilization effect observed in this work is not entirely explained. As mentioned before, the signal stability is achieved by the renewal of the electrodic surface during the negative polarization of the electrode, by potential cycling (cyclic voltammetry), or by reactivation pulses at −1.50 V (proposed method). The ability of MPA to restrain the fouling effect in very complex systems has already been documented [32]. Still, more research must be carried out to elucidate the accurate signal loss mechanism and the reactivation process presented in this work.

### 2.3. Analytical Parameters

**Figure 3:**
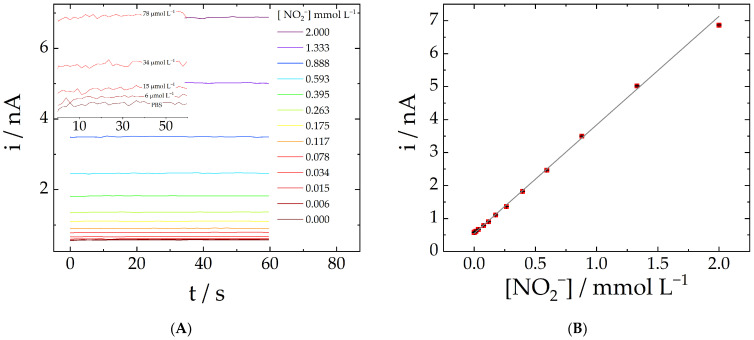
(**A**) Amperograms recorded with a carbon fiber microelectrode in PBS at varying concentrations of nitrite using the proposed protocol. Inset: Magnified amperograms obtained at low concentrations. (**B**) shows the calibration plot (R² = 0.998 and sensitivity = 3.23 nA L mmol^−1^).

At the optimized conditions, a linear calibration plot for nitrite was obtained with the three-pulse amperometric method in the 0.7 to 92 mg L^−1^ concentration range (15 to 2000 µmol L^−1^), with a correlation coefficient of 0.998. The limit of detection (LOD) and limit of quantification (LOQ) values were found to be 0.25 and 0.84 mg L^−1^, respectively (5.5 and 18 µmol L^−1^), calculated using the literature definition (3σ/s and 10σ/s, respectively, in which σ corresponds to the standard deviation of the background (four measurements) and s corresponds to the sensitivity (slope of the analytical plot (Figure 3B)).

Figure 3A shows the amperograms obtained for different nitrite concentrations in PBS using the three-pulse amperometric approach. The inset shows the responses corresponding to the lowest concentrations: 0 (only PBS), 6, 15, 34, and 78 µmol L^−1^. The calibration plot is presented in Figure 3B.

**Figure 4:**
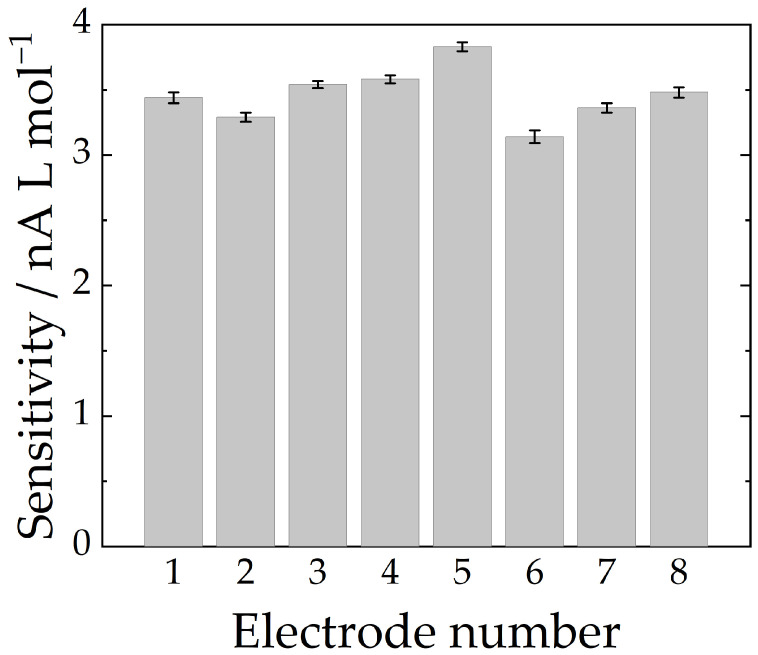
Sensitivity dispersion among repetitions. Error bar calculated as the standard deviation of the slope in the linearization. Mean sensitivity = 3.4 ± 0.2 nA L mol^−1^.

A series of calibration plots were obtained on different days with freshly polished carbon fiber microelectrodes, and the relative standard deviation of the slope values was found to be 6.4%, n = 8. Such RSD value can be considered as an indication of the reproducibility—i.e., the inter-day accuracy with some experiments conducted by different operators. The repeatability was assessed by calculating RSD values corresponding to seven consecutive measurements in four different nitrite solutions. Table 2 brings the mean, standard deviation, and RSD values for nitrite solution in various concentrations. Figure 4 presents the sensitivity dispersion.

The proposed method presented a good set of analytical parameters compared to other methods based on bare electrodes. Table 3 shows a brief comparison between various methods for nitrite determination. Although unsuitable for extremely low concentrations, the proposed method can be employed in many applications, and its signal stability can benefit long-term measurements.

**Figure 5:**
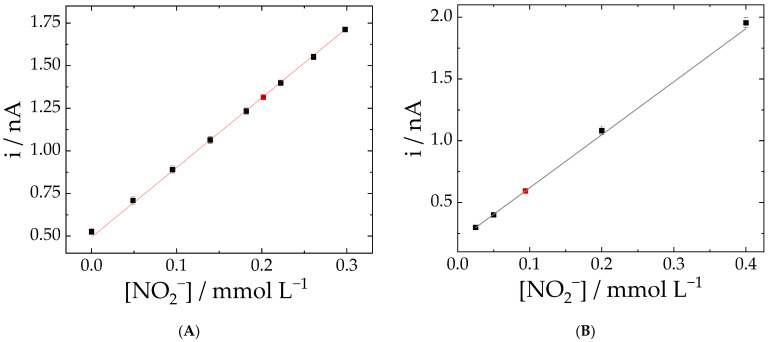
(**A**) Calibration plot prepared in artificial saliva. The red dots correspond to a fortified sample containing a known amount of nitrite. R² = 0.998 and sensitivity = 4.08 nA L mmol^−1^. (**B**) Calibration plot prepared in tap water. The red dots correspond to a fortified sample containing a known amount of nitrite. R² = 0.9998 and sensitivity = 4.47 nA L mmol^−1^.

The usefulness of the proposed approach was examined by measuring the nitrite content in synthetic saliva and tap water samples. Figure 5A shows the calibration plot prepared in synthetic saliva, and the red dot corresponds to a sample fortified with a known amount of nitrite (0.208 mmol L^−1^). The resulting concentration was determined as 0.202 mmol L^−1^, corresponding to a deviation of 2.6%. As a second application, a tap water sample was tested. The nitrite concentration in the tap water sample was below our detection limit, so the sample was fortified with 4.6 mg L^−1^ nitrite (0.1 mmol L^−1^). The concentration calculated with the calibration curve was in fair agreement with the fortification, with a 9% of deviation from the added amount of the target analyte. The calibration curve obtained for the tap water is presented in Figure 5B, and Table 4 shows the results obtained for both samples.

## 3. Materials and Methods

### 3.1. Chemicals, Materials, and Samples

All solid reagents were of analytical grade and used without further purification. Phosphate buffer saline was obtained from Merck (Darmstadt, Germany) as tablets, and the solutions were prepared using water obtained from a Nanopure Infinity System apparatus (Barnstead, Dubuque, 18.2 MΩ cm resistivity). Sodium chloride, potassium chloride, and carboxymethylcellulose (DS = 0.7) were obtained from Sigma Aldrich. Calcium chloride was obtained from Merck, and sodium hydrogen carbonate was obtained from QM (Mogi das Cruzes, Brazil).

#### 3.1.1. Synthetic Saliva Samples

The synthetic saliva sample was prepared with inorganic components and a thickener, as described in the literature [41]. Briefly, the synthetic saliva sample were prepared in ultrapure water with the following composition: 1.5 mmol L^−1^ CaCl_2_, 8.2 mmol L^−1^ NaHCO_3_, 4.8 mmol L^−1^ NaCl, 137 mmol L^−1^ KCl, and 4 mmol L^−1^ KH_2_PO_4_ and 0.5% of carboxmethylcellulose (DS = 0.7)

#### 3.1.2. Tap Water Samples

The tap water samples were obtained from the laboratory faucet and used without any further preparation.

### 3.2. Electrodes and Instrumentation

An Autolab PGSTAT128 (Eco Chemie, Utrecht, Netherlands) with data acquisition software made available by the manufacturer, Nova version 1.11 (Metrohm Autolab, Utrecht, Netherlands) was used for electrochemical measurements. Experiments were performed in a conventional electrochemical cell using a Ag|AgCl (saturated KCl) electrode, a graphite cylinder as a counter electrode, and a homemade carbon fiber microelectrode as the working electrode.

The carbon fiber microelectrode was fabricated as described in the literature [42]. Briefly, a 10 µm diameter carbon fiber (Solvay, Brussels, Belgium) was fixed in a NiCr wire with a conductive silver epoxy resin. The carbon fiber was then sealed with an insulating epoxy resin inside a plastic pipette tip. The resulting microelectrode was then polished with sandpaper and the electrochemical response was evaluated in a K_3_[Fe(CN)_6_] + KCl solution.

## 4. Conclusions

In summary, performing a pulsed amperometry technique can highly enhance the signal stability for the nitrite oxidation process carried out at a carbon fiber microelectrode. The developed method presented a good antifouling effect, with less than 1% signal variation after 20 min. The calibration plot exhibited a good linearization (R² = 0.998). The LOD and LOQ were determined in 5.5 and 18 µmol L^−1^, respectively, with a linear range from 6 to 2000 µmol L^−1^.

## Figures and Tables

**Table 1:** Nitrite oxidation current decay over 20 min for voltammetric and amperometric techniques.

Technique	Signal Change after 20 min
Amperometry	−47.7%
Differential pulse voltammetry	−35.5%
Cyclic voltammetry	−23.8%
Extended cyclic voltammetry	−0.35%
Proposed method	0.86%

**Table 2:** Repeatability study performed at varying nitrite concentrations (n = 7).

Nitrite Concentration/mmol L^−1^	Mean/nA	Standard Deviation/nA	RSD
0.25	1.334	0.004	0.4%
0.40	1.579	0.003	0.2%
0.80	2.611	0.004	0.2%
2.00	5.89	0.01	0.2%

**Table 3:** Comparison of analytical parameters for various methods for nitrite detection using bare electrodes.

Material	Linear Range (µmol L^−1^)	LOD (µmol L^−1^)	Technique	Ref.
Bare CFE	6–2000	5.5	MPA	This work
Bare GCE	50–4700	200	CV	[35]
	20–6400	300	DPV	
	2.5–10	0.4	Amp.	
Bare Pt	5–1000	2	Amp.	[23]
Bare Au	10–600	0.65	DPV	[36]
CGE/Zn-TPPS	<1000	0.1	FIA-Amp.	[37]
CGE/TiTaPc	<3500	1	CV	[38]
Au/MWCNTs/MoS_2_	12–2100	4.0	Amp.	[39]
Au/NPG	1–1000	0.01	Amp.	[18]
AgNC@NCS	1–1400	0.38	DPV	[40]

CFE: carbon fiber microelectrode; GCE: glassy carbon electrode; Zn-TPPS: meso-tetra (4-sulphonatephenyl) porphyrinate; TiTaPc: titanium tetraamino phthalocyanine; MWCNTs: multi-walled carbon nanotubes; NPG: nanoporous gold; AgNC: silver nanocluster; NCS: nitrogen-doped carbon. MPA: multiple pulse amperometry; CV: cyclic voltammetry; DPV: differential pulse voltammetry; Amp.: amperometry; FIA: flow injection analysis.

**Table 4:** Nitrite content in tap water (n = 3) and synthetic saliva (n = 4) determined by using the three-pulse amperometric method.

Sample	Fortification mg L^−1^ (µmol L^−1^)	Concentration Recovered mg L^−1^ (µmol L^−1^)	Deviation
Tap water	4.6 (100)	4.1 ± 0.1 (91 ± 3)	8.9 %
Synthetic saliva	9.6 (208)	6.9 ± 0.2 (202.5 ± 0.8)	2.6 %

## Data Availability

Data will be made available on request.

## Supplementary Materials

The following supporting information can be downloaded at: https://www.mdpi.com/article/10.3390/molecules28010387/s1, Figure S1: Consecutive cyclic voltammograms recorded with a carbon fiber microelectrode in PBS. Figure S2: (A) Signal decay after 20 minutes and reactivation potential correspondence. (B) Current variation in 20 minutes and reactivation time step correspondence. Figure S3: (A) Mean current and stabilization potential correspondence. (B) Current variation in 20 minutes and stabilization time step correspondence. Figure S4: (A) Mean current and oxidation potential correspondence. (B) Mean current and oxidation time step correspondence.
