# Supplementary material for: Multiple Pulse Amperometry—An Antifouling Approach for Nitrite Determination Using Carbon Fiber Microelectrodes
Source: Molecules. 2023 Jan 2;28(1):387. doi: 10.3390/molecules28010387 (PMC9824076; doi:10.3390/molecules28010387)
Supplement: Supplementary file 1 [file molecules-28-00387-s001.zip › molecules-2094211-supplementary.pdf]

Supporting Information

Multiple pulse amperometry, an antifouling approach for nitrite determination using carbon fiber microelectrodes

Douglas P. M. Saraiva, Daniel V. Braga, Bruna Bossard, Mauro Bertotti1 *

Department of Fundamental Chemistry, Institute of Chemistry, University of São Paulo, Av. Prof. Lineu Prestes, 748, 05513-970, São Paulo - SP, Brazil.

*Correspondent author: mbertott@iq.usp.br (Mauro Bertotti), Tel.: +55 11 3091-2694

The current decrease in PBS and in nitrite solution.

As stated in the paper, the current decreases during the cycles in CVs recorded in solutions containing nitrite (2 nA at 0.825V after 30 cycles), and such an effect is caused by the poisoning and passivation of the electrode. A much less significant influence is noticed in PBS, as the current decrease is only about 10 pA, as can be seen in Figure 1. This confirms that the poisoning of the electrode surface is attributed to nitrite oxidation products.

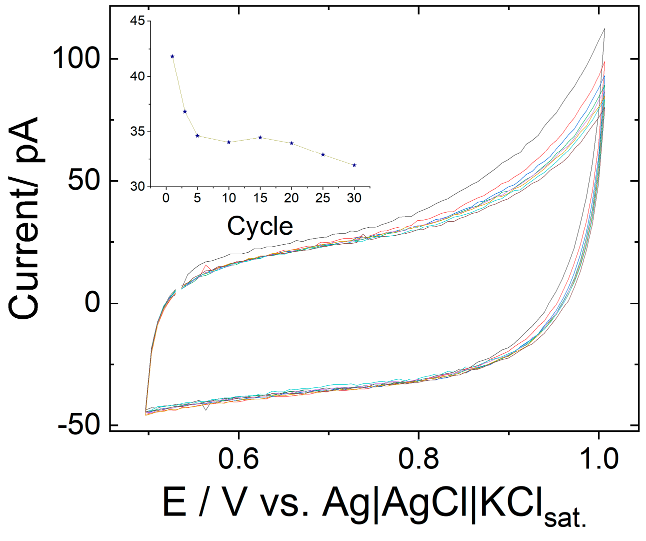

Figure S1. Consecutive cyclic voltammograms (n = 30) recorded with a carbon fiber microelectrode in PBS. Inset: Current decrease at 0.825V over the cycles.

Multiple pulse amperometry, parameter optimizations.

Three couples of parameters were optimized. The first couple was the reactivation potential and time step, as shown in Figure S2.

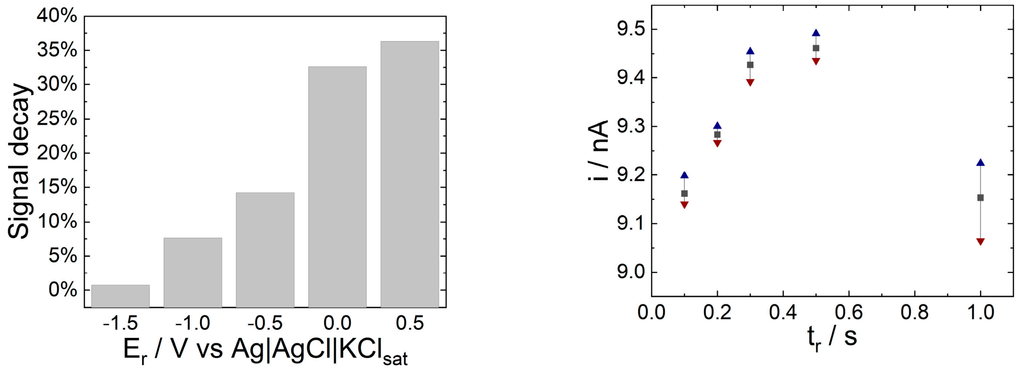

(A) (B)

Figure S2. A) Signal decay after 20 minutes and reactivation potential correspondence. (B) Current change after 20 minutes and reactivation time step correspondence.

The second couple of parameters to be optimized was the stabilization potential and time step, as shown in Figure S3.

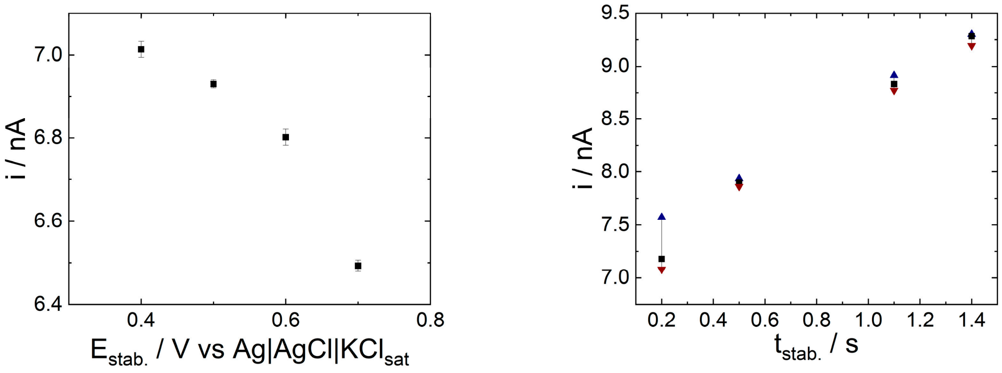

(A) (B)

Figure S3. A) Mean current and stabilization potential correspondence. (B) Current variation at 20 minutes and stabilization time step correspondence.

The last couple of parameters to be optimized was the oxidation potential and time step, as shown in Figure S4.

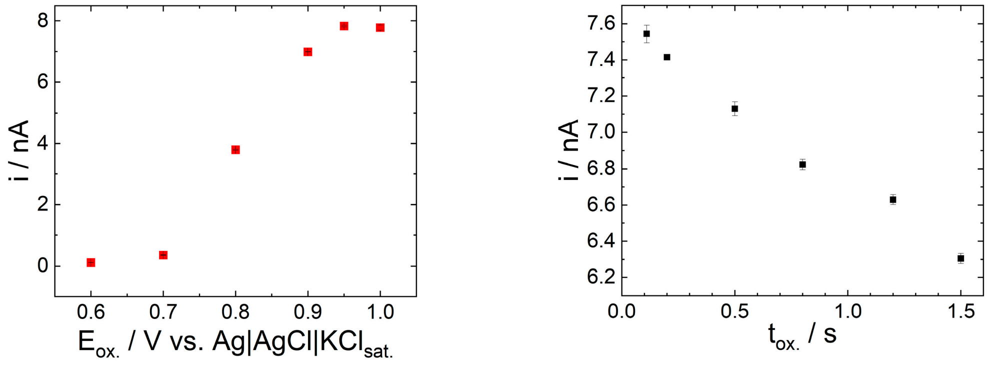

(A) (B)

Figure S4. A) Mean current and oxidation potential correspondence. (B) Mean current and oxidation time step correspondence.
